# Future Directions in Cardiac Amyloidosis

**DOI:** 10.14797/mdcvj.1071

**Published:** 2022-03-14

**Authors:** Barry Trachtenberg

**Affiliations:** 1 Houston Methodist Heart & Vascular Center, JC Walter Houston Methodist Transplant Center, Houston Methodist Hospital, Houston, Texas, US

**Keywords:** cardiac amyloidosis, transthyretin cardiomyopathy, light-chain amyloidosis, anti-fibril therapy

## Abstract

Just a few years ago, cardiac amyloidosis (CA) was rarely diagnosed. With poor treatment options and delayed and infrequent diagnoses, most patients who were eventually recognized to have CA were referred for hospice care. Now, the availability of sponsored genetic testing, increased use of nuclear scintigraphy, and widespread recognition have contributed to an increasing number of patients being diagnosed with transthyretin amyloid cardiomyopathy (ATTR-CM). Concomitantly, with the increased recognition of concurrent conditions (eg, carpal tunnel syndrome, lumbar stenosis, and low-flow, low-gradient aortic stenosis), specialists such as orthopedic surgeons and structural cardiologists are increasingly involved in diagnosing ATTR-CM.

Although the majority of patients are still being diagnosed either too late or having their diagnosis missed altogether, we have entered an exciting new era in the treatment of cardiac amyloidosis with improved diagnostic tools, disease recognition, and different therapeutic options for both ATTR and light-chain amyloidosis (AL). As a result, survival is improving, and we are no longer faced with a dualistic choice between hospice or organ transplant. The future goal is to develop anti-fibril therapies that will be safe and effective at removing deposited amyloid fibrils and restoring organs to their pre-amyloid state. For the millions of carriers of variant ATTR, enhanced testing followed by genetic editing may allow a cure even before patients develop clinical signs of the disease.

## Introduction

During a week of cardiology consultation during my first year of fellowship, our service diagnosed two patients with cardiac amyloidosis. This was a rarity at the time. One patient had transthyretin amyloidosis (ATTR) and the other light chain amyloidosis (AL). Neither were eligible for heart transplant due to age and extracardiac manifestations, respectively. Having no other options to treat them apart from diuretics, both were quickly dispatched to home hospice. Soon thereafter, my senior fellow told me excitedly of the recent literature showing feasibility and reasonable outcomes of patients with AL having heart transplant followed by autologous stem cell transplant. We were fascinated and hopeful that we might have options beyond simply telling patients to get their affairs in order.

Flash forward a dozen years, and one can only marvel at the incredible achievements in the recognition, diagnosis, and treatment of cardiac amyloidosis. Once an orphan disease, now there is a wave of interest in diagnosing amyloid, and the story of amyloidosis serves as a roadmap that those who treat other rare diseases strive to follow.

In this special issue of the journal, we have learned that ATTR is more prevalent than previously thought.^1^ The availability of sponsored genetic testing programs, rising use of nuclear scintigraphy, and increased recognition have all led to an increasing number of patients being diagnosed with ATTR cardiomyopathy (ATTR-CM). Simultaneously, with the increased recognition of concomitant conditions (eg, carpal tunnel syndrome, lumbar stenosis, and low-flow, low-gradient aortic stenosis), orthopedic surgeons and structural cardiologists are increasingly involved in diagnosing ATTR.

**Figure 1:**
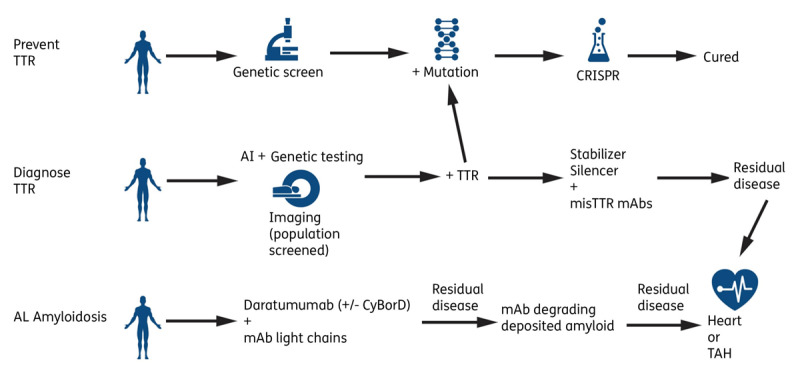
Potential future directions in cardiac amyloidosis. TTR: transthyretin; mAb: monoclonal antibody; CRISPR: clustered regularly interspaced short palindromic repeats; TAH: total artificial heart; AL: light chain amyloidosis; AI: artificial intelligence

Despite these advances, the majority of patients are still being diagnosed either too late or not at all. To make inroads into disease recognition, two criteria are essential. First is the use of machine learning to create algorithms from electronic health data, which will connect the dots of multiple comorbidities; fledgling efforts have already been made in this area (***Figure 1***).^2^ Second and most importantly, primary care providers, as well as general cardiologists and neurologists, need more education on the red flags, epidemiology, and diagnostic pathways of amyloidosis. This last criterion remains a major challenge as a large education deficit continues to persist.

## Diagnosis and Treament of Attr

In terms of the diagnosis of ATTR, the retooled use of nuclear scintigraphy and sponsored genetic testing programs have been instrumental in providing a noninvasive diagnostic tool and helping with cascade screening of family members, respectively. Cardiac magnetic resonance (CMR) imaging is increasingly being used for diagnosis (as well as diagnosis and prognosis of cardiomyopathies, viability assessment, or preprocedural planning). CMR often leads to the incidental diagnosis of cardiac amyloidosis, although it is unable to distinguish amyloid types. The use of novel tracers using positron emission tomography (PET) have shown promise in not only diagnosing amyloid but also differentiating the types of amyloidosis.^3^ It is likely that PET imaging, in addition to nuclear scintigraphy and MRI, will be a very useful tool for diagnosis and serial monitoring of response to treatment.

As more of the population uses direct-to-consumer DNA testing that includes screening for genetic diseases, more people will be made aware that they are carriers of variant TTR. Given that 3% to 4% of Black Americans are carriers of the Val122Ile variant (originating in western Africa),^4^ the most common variant in the United States, increased recognition followed by cascade screening of relatives may lead to enhanced screening of carriers and thus earlier diagnoses.

The treatment of ATTR has gone from having zero medications approved by the US Food and Drug Administration (FDA) before 2017 to three drugs currently on the market. In addition, several more therapies—including new TTR stabilizers and RNA silencers—are being investigated for the treatment of both wild type and hereditary ATTR cardiomyopathies, as summarized in the article by Stern and Patel in this issue.^5^ Other therapies under investigation include monoclonal antibodies (mAB) against misfolded TTR protein (misTTR mAbs). These anti-TTR antibodies are able to selectively bind to monomeric, misfolded TTR but not to native TTR, and they can both stabilize and degrade TTR fibrils by inhibiting the fibrillogenesis process. Currently, these misTTR mAbs have showed promise in phase I study subjects with ATTR-CM, and additional studies are being planned.^6,7^

Finally, in one of the more remarkable feats not only in amyloid treatment but also in modern science altogether, six patients with hereditary ATTR polyneuropathy had their mutations genetically edited using clustered regularly interspaced short palindromic repeats and associated Cas9 endonuclease (CRISPR) technology.^8^ Administered in a single dose, a new in vivo gene-editing therapeutic agent called NTLA-2001 was able to achieve a targeted knockout of TTR. If ongoing and long-term studies prove to be as successful, hereditary ATTR may actually be cured in our lifetime! In fact, with treatment and potential cures on the horizon, it is not far-fetched to imagine screening at-risk populations for at-risk mutations in early adulthood.

## Progress in Light Chain Amyloidosis Treatment

In addition to progress made in the treatment of ATTR, there also have been major advances in the treatment of light chain amyloidosis (AL). Since 2008, treatment with proteasome inhibitors such as bortezomib have had a major impact on long-term survival in patients with AL amyloidosis.^9^ Another major game-changer in AL treatment occurred in 2021 when daratumumab became the first FDA-approved treatment for AL amyloidosis.^10^ Daratumumab is an anti-CD38 human IgG monoclonal antibody agent administered subcutaneously to target the CD38 antigen overexpressed on clonal plasma cells. Daratumumab has excellent short-term organ response, and long-term data will be highly anticipated. Another anti-CD38 monoclonal antibody currently under investigation, isatuximab, has different binding epitopes on the CD38 antigen compared to daratumumab.

In AL amyloidosis, immunoglobulin heavy chain translocation t(11;14) is the most common cytogenic aberration and leads to overexpression of the BCL-2 protein, which suppresses apoptotic pathways.^11^ Future trials aim to study oral B-cell lymphomas 2 inhibitors (ie, venetoclax) in patients with t(11;14) translocation.

While current therapies are excellent at turning off the spigot that produces light chains, there is an unmet need for therapies that remove existing amyloid fibrils already deposited in organs, most critically the heart. This is a critical need given the oft-delayed recognition and diagnosis of amyloidosis until there is too much fibril deposition in the heart and other organs. Therefore, in addition to monoclonal antibodies being developed against misfolded TTR, monoclonal antibodies that target specific epitopes of misfolded light chains have also been developed and are being studied. Birtamimab (formerly NEOD001) is a humanized form of murine monoclonal antibody 2A4, which binds to an epitope on the misfolded light chain protein to target AL amyloid deposits in organs. While an initial phase III trial (The PRONTO Study) did not show benefit, post-hoc analysis showed a possible survival benefit in the most advanced patients (ie, those with Mayo Stage IV), and thus a new trial is under investigation in these patients.

CAEL-101 (11-1F4) is an IgG1 monoclonal antibody that binds to a cryptic epitope at the N-terminal of misfolded kappa and lambda light chain protein.^12^ Ongoing phase III studies are currently evaluating CAEl-101 on top of background CyBorD therapy for patients with treatment-naïve Mayo Stage IIIa AL and Stage IIIb cardiac amyloidosis.

## Transplantation in Amyloidosis

The role of transplantation in cardiac amyloidosis remains challenging. First, the benefit of autologous stem cell transplant (ASCT) in AL amyloidosis is not entirely clear due to a dearth of randomized controlled studies in this population.^13^ However, given that newer therapies (eg, daratumumab) are achieving a hematological response and often an organ response as well, one must wonder if there will be a need for ASCT in the future. Second, for AL amyloidosis, deciding who is a candidate for transplant and which patients with advanced cardiac amyloidosis can improve with current chemotherapies remains a significant clinical dilemma, especially with the increased risk of sudden cardiac death in these patients. Hopefully, collecting multicenter registry data will help clarify prognostic factors that might aid in these decisions. While staging with cardiac biomarkers and free light chains (eg, Mayo staging, European revised staging) has proven to be incredibly useful,^14^ there is a fundamental need for additional parameters to enhance prognostication, such as imaging data, hemodynamics, novel serum biomarkers, or other clinical markers.

For ATTR-CM, heart, liver, or combination heart-liver transplants have been performed for patients with advanced disease. For hereditary ATTR-CM, a liver transplant has been performed not because of intrinsic liver damage but because it is the primary source of the mutated TTR. Despite liver transplant, extracardiac manifestations of TTR (especially neurological) progress after transplant; this is due, in part, to the “seeding” effect of amyloid fibrils, in which the liver responds to the presence of any TTR fibrils in the body by producing new wild-type TTR fibrils.^15^ Presently, many patients need to take RNA silencers or TTR stabilizers after heart-liver transplant to decrease progression of extracardiac amyloidosis. As therapies that decrease TTR production improve, it is likely that liver transplantation may not be necessary, and thus many centers have stopped offering them for TTR. Finally, as other therapies such as mAbs, which can degrade already deposited fibrils, or CRISPR technology come to fruition, one can envision a future where patients may not need heart transplantation as part of their therapeutic options.

## Conclusion

We have entered an exciting new era in the treatment of cardiac amyloidosis. There are improved diagnostic tools, increased disease recognition, and several different treatment options for both ATTR and AL amyloidosis. Survival is improving, and we are no longer faced with dualistic choices between hospice or organ transplant. Despite these incredible advances, too many patients are diagnosed late, when deposition of amyloid fibrils in the heart and other organs is too much to overcome. In the future, we are hopeful that antifibril therapies for both ATTR-CM and AL will be safe and effective at removing deposited amyloid fibrils and restoring organs to their pre-amyloid state. For the millions of carriers of variant TTR, enhanced testing followed by genetic editing may allow a cure even before patients develop clinical signs of the disease.

Instead of telling newly diagnosed cardiac amyloid patients to start writing their wills, I can now offer many of them treatments that can prolong survival considerably and improve their disease burden and quality of life. I ask my patients to stop googling “cardiac amyloidosis” and stop reading outdated, doom-filled articles that inform them that they will die within 6 months. Although, tragically, we still lose too many patients to sudden cardiac death, we can offer hope for most patients. Thanks to dedicated scientists, clinicians, and patient participation in research, we have come an incredibly long way in the past 10+ years in the treatment of cardiac amyloidosis, and the future looks brighter than ever before.

## Key Points

- Genetic screening will increase early recognition of hereditary transthyretin amyloidosis (ATTRh).
- Artificial intelligence/machine learning and advances in imaging will increase recognition of cardiac amyloidosis.
- ATTRh may be cured in our lifetime due to CRISPR (clustered regularly interspaced short palindromic repeats) technology.
- Monoclonal antibodies against amyloid proteins and fibrils may add to the growing armamentarium of treatments available to treat cardiac amyloidosis.
